# A Survey of the Practice *Status Quo* of Ultrasound-Guided ECC Tip Location for Neonatal Patients in 31 Provinces of China

**DOI:** 10.3389/fped.2022.879920

**Published:** 2022-07-14

**Authors:** Xuexiu Liu, Xiaojun Tao, Ye Xu, Xianhong Zhang, Liping Wu

**Affiliations:** ^1^Department of Neonatology, Children's Hospital of Chongqing Medical University, Ministry of Education Key Laboratory of Child Development and Disorders, National Clinical Research Center for Child Health and Disorders, China International Science and Technology Cooperation Base of Child Development and Critical Disorders, Chongqing Key Laboratory of Pediatrics, Chongqing, China; ^2^Radiology Department, Children's Hospital of Chongqing Medical University, Chongqing, China; ^3^Department of Nursing, Children's Hospital of Chongqing Medical University, Chongqing, China

**Keywords:** neonate, ECC, ultrasound, cross-sectional study, China

## Abstract

**Objective:**

To investigate the *status quo* of implementing ultrasound (US)-guided epicutaneo-caval catheters (ECC) tip location for neonatal patients in 31 provinces.

**Methods:**

The convenience sampling method was used to investigate the nursing managers and ECC (or intravenous therapy) nurses of 91 hospitals in 31 provinces from October 29 to November 10, 2021.

**Results:**

The survey involved a total of 182 medical staff, including 91 managers and 91 nurses, and 91 institutions, including 22 children's hospitals, 49 general hospitals and 21 maternal and child health care hospitals. Sixteen hospitals (17.6%) carried out US-guided ECC for neonatal patients; 176 subjects (96.7%) of the 91 hospitals had known about or heard of the technology of US-guided ECC. The low awareness of operators of the tip location of ECC catheters in children under ultrasound guidance (OR = 2.690, 95% CI = 1.163–6.221), limited conditions in existing wards (OR = 2.953, 95% CI = 1.285–6.790), and insufficient funds (OR = 2.836, 95% CI = 1.149–7.004) were the independent risk factors responsible for the failure to carry out ultrasonic-guided location of ECC tips in newborns.

**Conclusion:**

The popularity of neonatal US-guided ECC location was seriously hindered by factors such as a low awareness rate of the project, the low qualification certification rate of the nursing staff, a flawed performance allocation system, and the lack of a professional team, among other factors.

## Introduction

Epicutaneo-caval catheters (ECCs) have been widely used in neonatal intensive care units (NICUs) for intravenous nutritional support and long-term drug infusion (1). Maintaining the catheter tip within the superior or inferior vena cava is critical because malposition may induce adverse outcomes (2, 3). Clinically, chest radiography (CR) has been used as the “gold standard” to confirm the site of the catheter tip (4, 5). However, accumulating evidence has shown the drawbacks of tip positioning by CR, including special requirements for the imaging room, retrospective imaging, ionizing radiation, and a time-consuming operation. Comparatively, ultrasonography (US) has many virtues, such as allowing for an easier operation at bedside, using non-ionizing radiation, and allowing real-time imaging and direct visualization of the catheter tip and cardiovascular structures. In fact, it has recently been used, with success, for the evaluation of ECC location in adult and child patients (6, 7). However, as a new and promising technology, there is still little known about its application in neonatal medical centers. Few reports have focused on this topic. For this reason, this study conducted a cross-sectional survey on the practice status of neonatal US-guided PICC placement at 91 hospitals in 31 provinces and municipalities in China, aiming to provide detailed data on the application of this project and to better understand the factors affecting its application.

## Methods

### Participants

The study involved the neonatal wards of 91 secondary and tertiary hospitals in 31 provinces and municipalities, including Beijing, Tianjin, Shanghai, Chongqing, Zhejiang Province, Jiangsu Province, Guangdong Province, Sichuan Province, etc. The convenience sampling method was used and for each hospital, a neonatal nursing director and a neonatal ECC team leader were selected as the respondents between Oct 29, 2021 and Nov 10, 2021. Finally, a total of 182 nursing staff were enrolled in the survey. The nurses included were the neonatal ECC team leader and the head nurse of the department. Exclusion criteria were as follows: (1) those who did not gain the nursing practice qualification certificate; (2) those who were not currently working; (3) those who did not receive a minimum of a college education; and (4) those who were undergoing further education, regular training or an internship. The institutional review board approved the study (Approval No., 2021-342).

### Survey Tools

A self-designed questionnaire was used. The first version of the “questionnaire for neonatal ECC tip ultrasonic location” was formed by reading relevant literature and integrating expert correspondence advice and after several rounds of discussion and modification by the research group. Before the formal survey, six nursing managers and 10 ECC specialist nurses were selected for the presurvey. The questionnaire was further modified according to the feedback in the form of expert group discussion. Then, an expert meeting was convened. Six experts in the field of ECC nursing and tool construction (with a minimum job status of supervisor nurse, a minimum of a bachelor's degree, at least 10 years of work experience and five or more years of experience in ECC) were invited to modify the questionnaire and finally form the final version, including 22 items.

The questionnaire consists of two parts: (1) Basic information questionnaire, including hospital name, department, region, hospital grade, hospital type, gender, age, education, professional title, position, how many years the respondents had worked, whether the respondents were ECC specialist nurses, etc. (2) Questionnaire on the development and management of neonatal ECC tip ultrasound location, including the ECC management mode of the hospital, whether they know and support the development of neonatal ECC US tip location, whether they have participated in a systematic training of neonatal ECC US tip location, whether they have the knowledge and ability to carry out neonatal ECC US tip location, their viewpoints to the advantages and disadvantages of neonatal ECC US tip location, whether the department/hospital has carried out neonatal ECC US tip location, whether there is a management system, workflow, emergency plan, effect evaluation and incentive policy for the main participants, personnel training methods, qualification certificate and operator access conditions, the main reasons for not carrying out neonatal ECC US tip location and which aspects can promote the development of the practice of neonatal ECC tip ultrasound location. The questionnaire was verified through content validity. The content validity was evaluated by two doctors and two nurses from neonatal wards, and two ECC specialist nurses and two intravenous therapy specialist nurses (with working experience ≥ 15 years). The content validity index of the questionnaire was 0.92.

### Data Collection Methods

The survey was conducted by relying on several national communication platforms. The platforms include the pediatric nursing group members of the Chinese Medical Association, the national intravenous therapy exchange and learning platform, the ECC special class exchange group, the national pediatric special class exchange and learning group and others. One-to-one unified guidance was adopted to explain to the group members about the survey purposes, contents, respondents, and inclusion and exclusion criteria. Then, the questionnaire star link was issued, and the qualified respondents were invited to complete the questionnaire. Quality control methods were as follows: (1) anonymous questionnaire; (2) setting an IP address limit to ensure a respondent can only answer once; (3) setting required questions and using skip logic to ensure that a questionnaire with missing answers cannot be submitted; (4) manual checking to eliminate the questionnaires with regular options; and (5) screening according to the name of the hospital and asking that the head nurse of each hospital and a ECC team leader that he or she recommended fill in the questionnaire to ensure only that two questionnaires were recovered from the hospital. Finally, a total of 182 valid questionnaires were collected.

### Statistics

The data acquired from the questionnaire were imported into Excel software. After checking and sorting by two researchers, the data were imported into SPSS 26.0 for statistical analysis. The measurement data were described by means ± standard deviation, while the counting data were described by frequency, constituent ratio and percentage. Binary logistic regression analysis was used to analyze the factors affecting the hospital practice of neonatal ultrasound positioning. *P* < 0.05 was considered statistically significant.

## Results

### General Information About the Hospitals

The general information about the 182 respondents is shown in Table 1. They belonged to 91 medical institutions. These hospitals are grouped by type into children's hospitals (22, 24.2%), women's and children's hospitals (21, 23.1%) and general hospitals (49, 53.9%) and grouped by level into tertiary hospitals (77, 84.6%) and secondary hospitals (14, 15.4%). Among them, only 16 (17.6%) tertiary hospitals, including 4 children's hospitals (4.4%), five women's and children's hospitals (5.5%) and seven general hospitals (7.7%), carried out US-guided ECC for neonates (Table 2).

**Table 1:** General information of respondents (*n* = 182).

**Item**	**Persons, number (%)**
**Gender**
Male	2 (1.1)
Female	180 (98.9)
**Age (years)**
25–29	20 (11.0)
30–39	116 (63.7)
40–49	41 (22.5)
≥50	5 (2.7)
**Education**
Specialty	8 (4.4)
Undergraduate	170 (93.4)
Master's degree	4 (2.2)
**Professional qualifications**
Nurse	1 (0.5)
Senior nurse	57 (31.3)
Supervisor nurse	95 (52.2)
Deputy chief nurse	25 (13.7)
Chief nurse	4 (2.2)
**Duties and roles**
Responsible nurse	49 (26.9)
Responsible maintenance team leader	40 (22.0)
Head nurse	90 (49.5)
Director of nursing department	1 (0.5)
**Working years (years)**
1–5	8 (4.4)
6–10	50 (27.5)
11–15	62 (34.1)
16–20	30 (16.5)
≥21	32 (17.6)
**ECC/intravenous specialist nurse**
ECC specialist nurse	95 (52.2)
Intravenous therapy specialist nurse	30 (16.5)

**Table 2:** Current situation of ultrasound localization of newborns in hospitals [Institutes (percentage,%)].

	**Item**	**Ultrasound localization of neonatal ECC tip (*n* = 16)**	**No ultrasound localization of neonatal ECC tip (*n* = 75)**	***P***
Hospital type	Children's Hospital	4 (25.0)	18 (23.7)	0.62
	General Hospital	7 (43.8)	42 (55.3)	
	Maternal and Child Health Hospital	5 (31.3)	16 (21.1)	
Hospital level	Second level (19)	0	14 (18.7)	0.12
	Tertiary (106)	16 (100.0)	61 (81.3)	

### Nurses' Recognition of Neonatal US-Guided ECC Tip Location

Of the 182 respondents, 176 persons (96.7%) expressed that they knew about neonatal US-guided ECC tip location, the other 6 (3.3%) did not; 172 persons (94.5%) expressed that they welcomed the implementation of neonatal US-guided ECC tip location; 134 persons (73.6%) expressed that they had attended systematic training or lectures on neonatal US-guided ECC positioning; 64 persons (35.2%) expressed that they held the knowledge to start the neonatal US-guided ECC positioning; 47 persons (25.8%) expressed that they had mastered the skills to start the neonatal US-guided ECC tip positioning.

### Scoring of the Advantages and Disadvantages of Neonatal US-Guided ECC Positioning

More intuition by dynamic display, easiness for operation, and reduced X-ray exposure ranked among the top three advantages of neonatal US-guided ECC positioning (Table 3). The disadvantages were increasing workload, increasing infection risk of patients, and increasing the medical disputes (Table 4).

**Table 3:** Advantage score of neonatal ultrasound localization (score, $\bar{x}$ ± s).

**Item**	**Score**
Dynamic display, more intuitive	4.99 ± 0.74
Easy to operate	4.91 ± 0.42
Reduce X-ray exposure	4.77 ± 0.43
The ectopic tip of the catheter was found and reset in time	4.70 ± 0.46
Position at any time	4.69 ± 0.47
Reduce operation time	4.45 ± 0.69
Cost reduction	4.31 ± 0.73

**Table 4:** Disadvantages score of neonatal ultrasound localization (score, $\bar{x}$ ± s).

**Item**	**Score**
Increase the workload of staff	2.85 ± 1.03
Increase the risk of infection in patients	2.69 ± 0.99
Increase the risk of medical disputes	2.55 ± 0.99

### Hospital-Executed Management of Neonatal US-Guided ECC Tip Location

Hospital-executed management of neonatal US-guided ECC tip location mainly includes the following aspects: main management contents, key participants, training methods, whether there is a corresponding qualification certificate, and authorized qualification certificate of professional technicians (Table 5).

**Table 5:** Management work of the hospital for the implementation of neonatal ultrasound localization [institute (%)].

**Item**	**Number of cases**	**Composition ratio/rate (%)**
**Main management contents**		
Formulate the management system and work flow of ultrasound positioning neonatal ECC	16	50.00
Formulate emergency plans related to ultrasonic localization of neonatal ECC tip	14	43.75
The effect of ultrasound localization of neonatal ECC tip was evaluated	22	35.48
Establishment of incentive policy for ultrasound localization of neonatal ECC tip	8	25.00
Someone or organization is responsible for coordinating and managing the ultrasonic localization of neonatal ECC tip	18	56.25
**Key participants**		
Licensed ECC specialist nurse	28	87.50
Qualified intravenous nurse	10	31.25
Ultrasound doctor	16	50.00
Clinician	14	43.75
**Training methods**		
Professional training course	20	62.50
Academic conference	18	56.25
Collective theory teaching and simulation demonstration	18	56.25
Other	8	25.00
**Whether there is a corresponding qualification certificate**		
Both	14	43.75
Some have	12	37.50
None	6	18.75
**Authorized qualification certificate of professional technicians**		
One-time authorization	18	56.25
Regular reevaluation and reauthorization	14	43.75

### Negative Factors Affecting the Practice of Neonatal US-Guided ECC Tip Location

The negative factors affecting the practice of neonatal US-guided ECC tip location are shown in Table 6. A binary logistic regression analysis was conducted with whether neonatal US-guided ECC tip location was carried out as the dependent variable and 11 negative factors affecting the implementation as independent variables. The variables were coded as yes = 1 and no = 0, and the values were plugged into the regression equation. The single-factor study showed that the negative factors were low operator awareness of neonatal US-guided ECC tip location, lack of skill mastery, limited ward conditions, lack of US-guided positioning regulations and insufficient funds. The variables with *P* < 0.05 were further studied using multivariate analysis with the stepwise method adopted to screen the variables. The results showed that low operator awareness of neonatal US-guided ECC tip location (OR = 2.690, 95% CI = 1.163–6.221), limited ward conditions (OR = 2.953, 95% CI = 1.285–6.790) and insufficient funds (OR = 2.836, 95% CI = 1.149–7.004) were independent risk factors (Table 7).

**Table 6:** Investigation of obstacles affecting the hospital to carry out neonatal ultrasound localization (*n* = 182).

**Item**	**Number of cases**	**Composition ratio/rate (%)**
**The existing ward conditions are limited such as equipment not purchased**		
Yes	125	68.7
No	57	31.3
**Poor technical mastery**		
Yes	123	67.6
No	59	32.4
**Operators have low awareness of the localization of ECC catheter tip in children under ultrasound guidance**		
Yes	110	60.4
No	72	39.6
**The ultrasonic localization system is not perfect**		
Yes	97	53.3
No	85	46.7
**The level of operators is uneven**		
Yes	95	52.2
No	87	47.8
**Lack of medical staff**		
Yes	84	46.2
No	98	53.8
**Insufficient policy support**		
Yes	85	46.7
No	97	53.9
**Insufficient fund**		
Yes	84	46.2
No	98	53.8
**Hospital managers lack understanding of this model**		
Yes	76	41.8
No	106	58.2
**The support of medical staff for this model is not high**		
Yes	70	38.5
No	112	61.5
**Patient's own reasons: such as vascular conditions, disease causes, etc**.		
Yes	67	36.8
No	115	63.2

**Table 7:** Logistic regression analysis of influencing factors of obstruction of neonatal ultrasound localization in hospital (*n* = 182).

**Variable**	**Univariate logistic regression**			**Multivariate logistic regression**		
	**Regression coefficient**	***P***	**OR (95% CI)**	**Regression coefficient**	***P***	**OR (95% CI)**
1. Operators have low awareness of the localization of neonatal ECC catheter tip under ultrasound guidance	0.944	0.021	2.571 (1.154–5.731)	0.989	0.021	2.690 (1.163–6.221)
2. The existing ward conditions are limited such as equipment not purchased	1.129	0.006	3.094 (1.388–6.898)	1.083	0.011	2.953 (1.285–6.790)
3. Insufficient fund	0.933	0.036	2.541 (1.065–6.063)	1.043	0.024	2.836 (1.149–7.004)
4. Poor technical mastery	0.866	0.033	2.378 (1.073–5.27)			
5. The ultrasonic localization system is not perfect	0.811	0.049	2.251 (1.002–5.054)			
6. Hospital managers lack understanding of the technology	0.455	0.279	1.576 (0.692–3.592)			
7. The support of medical staff for this model is not high	0.238	0.572	1.269 (0.555–2.899)			
8. The level of operators is uneven	0.426	0.29	1.532 (0.696–3.373)			
9. Insufficient policy support	0.136	0.735	1.146 (0.521–2.524)			
10. Lack of medical staff	0.467	0.257	1.596 (0.711–3.58)			
11. Patient's own reasons: such as vascular conditions, disease causes, etc.	0.79	0.088	2.203 (0.89–5.45)			

### The Respondents' Suggestions on Hospital Initiatives to Promote the Development of Neonatal US-Guided ECC Tip Location

One hundred fifty-three respondents (83.6%) thought that a special team was necessary to manage the organization, planning and implementation; 146 respondents (80.2%) thought that targeted training and evaluation was necessary; 145 (79.7%) suggested encouragement and support from hospitals or departments; 133 people (73.1%) suggested the establishment of relevant regulations and clarification of the workflow and responsibilities; 122 (67.0%) suggested strict implementation of hospital infection prevention and control and safety management; 117 (64.3%) report, statistics and analysis of ECC clinical data; and 107 (58.8%) suggested the development of the adverse event handling and reporting system.

## Discussion

ECCs have been used in Chinese health institutions for approximately 30 years. US-guided ECC placement was first introduced in the early 21st century but adopted much later for neonatal patients. Beginning last year, it was formally recommended in *Operation and Management Guidelines for Peripherally Inserted Central Catheter in Neonates* by the Neonatologist Society of Chinese Medical Doctor Association (8). To understand more about the practice *status quo* of neonatal US-guided ECC tip location in China, we launched this survey that involved 182 nursing managers and ECC team leaders from 91 hospitals in 31 provinces and municipalities in China. The survey demonstrated that only 16 tertiary hospitals (17.6%) have adopted neonatal US-guided ECC tip location. This may be due to the medical staff's unawareness of or reluctance to carry out novel services in neonates, more difficult imaging operations for the small blood vessels of neonates, and the lack of technology and management promotion.

In this study, 96.7% of the respondents expressed that they knew about neonatal US-guided ECC tip location; 94.5% supported it, and 73.6% had ever attended a training lesson. However, only 35.2% of the respondents hold the knowledge, and 25.8% hold sufficient skills to launch such an imaging projection. The low recognition of neonatal US-guided ECC tip location ranks among the three independent risk factors counteracting its popularization, which would mean that there are cause fewer chances to be trained in this technology. Therefore, it is necessary to strengthen publicity and promotion efforts of this technology (9, 10). Limited ward conditions and insufficient funds are also independent factors. The limited conditions of the ward mainly include the increased expenditure caused by the purchase of ultrasonic examination instrument, couplant and paper, as well as the training expenses caused by the training of professionals. According to our experience, domestic secondary hospitals are fully capable of purchasing Doppler ultrasound machines. The real causes of equipment shortages could be investment, bonus distribution, cost deductions, incentives and other institutional problems. Thus, we argued that project leaders should actively talk with hospital leaders to straighten out these interests. Insufficient human resources may also hinder the implementation of US in neonatal ECC catheterization, albeit no statistical significance was found in this study. Currently, the lack of medical nursing staff has become a global problem. Especially for neonatal specialist nurses, high work pressure and professional requirements have caused a critical shortage of workers (11). Therefore, we argue that it is essential that hospital leaders give full consideration to satisfying nurses' career demands, improving nurses' job satisfaction and reducing factors affecting nurses' career achievement, and establish a reasonable compensation mechanism, a scientific performance evaluation mechanism and a personnel salary system adapted to the attributes of the profession (12). Under the guidance of these beneficial systems, more nursing staff would be attracted to participate in pediatric and newborn nursing work.

The respondents agreed that the top three strategies to promote the application of neonatal US-guided ECC tip location were separately setting up a special team to organize, plan and implement the project (153, 83.6%), supplying systematic training for the people in the process (146, 80.2%), and gaining financial and institutional support from the hospital or department (145, 79.7%). Undoubtedly, the establishment of a set of targeted management systems at the hospital level would be greatly conducive to the development and continuous improvement of the project (13, 14), and ECC-specialized management would offer accurate and timely supervision and control throughout the ECC process, ensuring that the procedures are well-regulated to better reduce the risks (7, 15). Avoiding X-ray exposure, providing a real-time dynamic display, and the timely finding and reducing the chances of misplaced tips during catheter use rank among the top three merits of US-guided ECC tip location as recognized by the respondents. These merits of US have also been verified in publications (16–18). However, according to this study, neonatal US-guided ECC tip location is seldom used in Chinese medical institutions, and there are some deficiencies presented in the project's promotion and management. Therefore, it is suggested that the administrators and nursing experts of tertiary hospitals jointly formulate a unified standardized workflow and effectiveness evaluation index system for neonatal US-guided ECC tip location (19), construct clinical practice guidelines, and promote its standardization and scientific process (13, 14).

## Limitations

In this study, the questionnaire was distributed through the committee group of the pediatric nursing group of the Chinese Medical Association and other platforms, which may have led to fewer secondary hospitals being reached by the survey. In addition, the respondents of this study did not involve medical insurance policy-makers, medical insurance management departments or insurance companies, so the impact of medical insurance policy on technology popularization and development was not investigated.

## Conclusions

This study investigated the practice *status quo* of neonatal US-guided ECC tip location of 125 hospitals in 31 provinces, which revealed that the development of the project was seriously hindered by factors such as a low awareness rate of the project, the low qualification certification rate of the nursing staff, flawed performance allocation system, and lack of a professional team. It is suggested to strengthen the training, learning and qualification certification of medical staff, increase the sense of professional achievement of nurses, and establish a professional team to promote the publicity of neonatal US-guided ECC tip location and provide high-quality, standardized and scientifically-based services for neonatal patients.

## Data Availability Statement

The original contributions presented in the study are included in the article/supplementary material, further inquiries can be directed to the corresponding author/s.

## Ethics Statement

The institutional review board has approved the study: Approval No. 2021-342. Written informed consent was obtained from individual or guardian participants.

## Author Contributions

XL contributed to acquisition, analysis and interpretation of the data and acquisition, analysis and interpretation of the data and the drafting, and final approval of the manuscript. XT, YX, and XZ provided technical support and conceptual advice. LW designed the study. All authors made substantial contributions to the study and manuscript and met the criteria for authorship defined in the author instructions, read, and approved the final manuscript.

## Funding

This work was supported by the Chongqing Science and Technology Commission (2020MSXM028).

## Conflict of Interest

The authors declare that the research was conducted in the absence of any commercial or financial relationships that could be construed as a potential conflict of interest.

## Publisher's Note

All claims expressed in this article are solely those of the authors and do not necessarily represent those of their affiliated organizations, or those of the publisher, the editors and the reviewers. Any product that may be evaluated in this article, or claim that may be made by its manufacturer, is not guaranteed or endorsed by the publisher.
